# Rectal chloral hydrate sedation for computed tomography in young children with head trauma

**DOI:** 10.1097/MD.0000000000025033

**Published:** 2021-03-05

**Authors:** Quanmin Nie, Peiquan Hui, Haitao Ding, Zengwu Wang

**Affiliations:** a Department of Neurosurgery, Weifang People's Hospital, Weifang; b Department of Neurosurgery, Linyi Central Hospital, Yishui, Shandong, China.

**Keywords:** chloral hydrate, computed tomography, pediatric sedation, traumatic brain injury

## Abstract

Children evaluated in the emergency department for head trauma often undergo computed tomography (CT), with some uncooperative children requiring pharmacological sedation. Chloral hydrate (CH) is a sedative that has been widely used, but its rectal use for child sedation after head trauma has rarely been studied. The objective of this study was to document the safety and efficacy of rectal CH sedation for cranial CT in young children.

We retrospectively studied all the children with head trauma who received rectal CH sedation for CT in the emergency department from 2016 to 2019. CH was administered rectally at a dose of 50 mg/kg body weight. When sedation was achieved, CT scanning was performed, and the children were monitored until recovery. The sedative safety and efficacy were analyzed.

A total of 135 children were enrolled in the study group, and the mean age was 16.05 months. The mean onset time was 16.41 minutes. Successful sedation occurred in 97.0% of children. The mean recovery time was 71.59 minutes. All of the vital signs were within normal limits after sedation, except 1 (0.7%) with transient hypoxia. There was no drug-related vomiting reaction in the study group. Adverse effects occurred in 11 patients (8.1%), but all recovered completely. Compared with oral CH sedation, rectal CH sedation was associated with quicker onset (*P* < .01), higher success rate (*P* < .01), and lower adverse event rate (*P* < .01).

Rectal CH sedation can be a safe and effective method for CT imaging of young children with head trauma in the emergency department.

## 1 Introduction

Over 600,000 children are evaluated annually in American emergency departments (EDs) for head trauma.^[1]^ Computed tomography (CT) is the reference standard for emergently diagnosing traumatic brain injuries. Approximately 50% of these children undergo cranial CT scanning.^[2]^ CT scanning requires children to be as quiet and motionless as possible, but in young children, cooperation with these procedures is more difficult. After head trauma by accident, some children have increased nervous system excitability, often manifesting as fear, crying, or restlessness, thus increasing the difficulty of CT scanning.

Poor cooperation among children undergoing diagnostic procedures often necessitates the use of sedation or anesthesia to prevent movement and ensure optimal imaging quality. While anesthetic agents are frequently used to produce a uniform anesthetic response, to achieve this effect is time-consuming, resource-intensive, expensive, and not without risks^[3,4]^; thus, pure sedative rather than anesthetic agents is often adopted in the clinic. The major advantage of sedation is that it can also be performed in the ED.^[5]^

Prior research suggested that the type of sedative and route of administration are chosen depending on the child, the type of procedure, and the desired effect.^[6]^

The variability in sedation medications suggests a need for evidence-based guidelines.^[7]^ Little research has focused solely on head-injured patients sedated in the ED for cranial CT scanning.^[2]^

Because CT scanning is a nonpainful procedure and requires only the patient's immobilization, simple sedation can be used. Chloral hydrate (CH) is a sedative that is recommended in painless procedures in children who cannot cooperate with neurodiagnostic procedures, such as auditory brain stem response measurement and electroencephalography.^[8,9]^ The mechanism is that it mainly suppresses the ascending reticular activating system to produce nearly natural sleep. The results from earlier studies in ophthalmology indicated that CH might be an effective sedative in children, and it could be especially convenient in outpatient settings.^[10]^

Due to lack of cooperation and the small veins of children, injection (intravenous or intramuscular) is difficult and unfavorable. To some extent, the added pain caused by puncture reduces the sedative efficacy. Therefore, the parenteral pathway is not very suitable for children in the ED.^[11]^

CH can be administered orally or rectally.^[12]^ Due to the incomplete development of the gastrointestinal tract, brain injury and increased intracranial pressure, the incidence of nausea and vomiting in children with head trauma is relatively high.^[13,14]^ CH has a pungent taste, and oral administration could stimulate the upper gastrointestinal tract, resulting in nausea and vomiting^[15]^; thus, oral administration is inappropriate for children with head trauma. In our study, we used the rectal route instead of the oral route to avoid this gastrointestinal reaction. So far, there are many studies using oral CH for pediatric sedation, yet its use via the rectal route has not been well described.

Every sedative has adverse effects. Adverse outcomes are associated with all routes of drug administration and all classes of medication.^[16]^ Compared with other sedative agents, in addition to vomiting reactions, CH may have other adverse effects. Although previous studies demonstrated that the adverse effects included paradoxical agitation, motor imbalance, prolonged sedation, desaturation, hypotension and apnea,^[3]^ the rate and potential complications in our study may be different.

The purpose of this study was to evaluate the efficacy of rectal CH sedation by assessing the success rate of performing CT scanning in young children with head trauma and its safety by assessing the adverse effects related to sedation. We also sought to describe the time course of the sedative effect, determine the advantage of rectal CH in children sedation, and summarize our clinical experience.

## 2 Materials and methods

The clinical data from January 2016 to December 2019 were studied retrospectively. This study was approved by the Ethics Committee of the hospital in compliance with the Declaration of Helsinki. Informed consent was obtained from parents or legal guardians. To keep the personal data private, the information was kept secure.

The study enrolled children who required CT scanning for the purpose of evaluating head trauma but who could not cooperate with the procedure without sedation. Children who were sleeping, cooperative, comatose, or under general anesthesia were excluded from the study. Exclusion criteria also included unstable respiration, unstable circulation, and allergy to CH.

Presedation evaluation was performed before the procedure by a neurosurgeon and a pediatrician. The assessment included age, health history, physical examination, weight, airway, and other diagnostic tests. The 2 designated doctors planned the sedation and supervised all the procedures taking place in the ED. In medically complex cases, an anesthesiologist was asked to evaluate and monitor the patient.

After the consultation and evaluation, CH (10%) at a dose of 50 mg/kg body weight, doubled with normal saline, was prepared and administered by a trained nurse. The detailed enema process was as follows: The front end of the disposable tube was lubricated with liquid paraffin first. After extracting the prepared drug by the disposable syringe, the nurse connected the syringe to the other end of the tube, inserted the tube into the rectum gently, pushed the drug over, pulled out the tube, pinched both sides of the buttocks to the anus, raised the buttocks, and held this position for 5 minutes. If the child had a defecation reflex before the pushing of the drug, we let the child defecate first. In the oral CH group, CH was administered orally at the same dose of 50 mg/kg.

After the sedative was administered, the children were placed in a quiet room to fall asleep. In the whole procedure, children were monitored for oxygen saturation and heart rate with a pulse oximeter, and the respiratory rate was also observed. Suction, oxygen, and a resuscitation chart were immediately accessible. Parents or legal guardians were allowed to stay with their children. If the outcome was not effective 60 minutes after the first dose, a second dose was supplemented at 25 mg/kg body weight.

After the sedative took effect, the children were put on the CT examination bed, other parts of the body were protected by lead clothing, and then CT scanning was performed. After completion of the procedure, the parents or legal guardians were encouraged to stimulate and awaken the children. Children who did not wake after the procedure were further monitored, and all of them remained at the hospital until they returned to their previous state of consciousness. Resumption and stable vital signs were considered to be recovered in our study.

The obtained images were evaluated by a trained radiologist. If the patient fell asleep and remained stable during the examination, leading to a clear image, this was regarded as obviously effective; if the patient basically remained still during the examination and the obtained image did not affect the diagnosis, this was regarded as basically effective; if the patient didn’t cooperate and could not complete the examination, which made the image lose diagnostic value, this was regarded as a sedative failure.^[17]^

The collected data were processed with GraphPad Prism 5 software (GraphPad Software, Inc., San Diego, CA). Continuous variables were presented as means and standard deviations (SD). Independent *t* tests were used to compare continuous data, *χ*^2^ tests or Fisher exact tests were used to compare categorical data, and paired *t* tests were used to compare differences in vital signs from before to after sedation. A value of *P* < .05 was taken as denoting statistical significance.

## 3 Results

There were 1603 children aged 5 to 40 months underwent CT examination from January 2016 to December 2019 in our hospital. The study group was made up of 135 (8.4%) children in the same period, aged 5 to 40 months, with a mean age of 16.05 (SD: 7.56) months and a median age of 15 months; 43.0% were ≤12 months, 57.0% were >12 months, 68.1% were boys, and 31.9% were girls.

**Figure 1:**
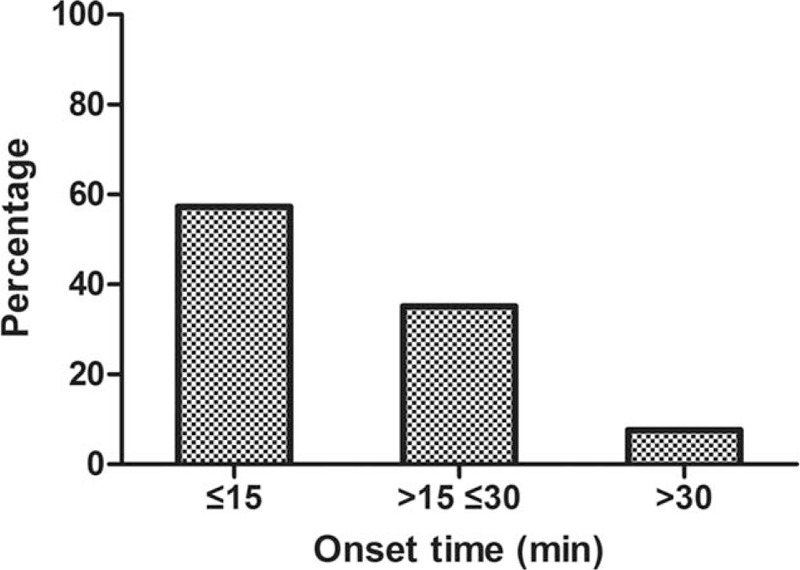
Different onset time of rectal CH sedation with the corresponding percentages of patients. A total of 57.3% patients showed a sedative effect at ≤15 min, 35.1% at >15 but ≤30 min, and 7.6% at >30 min. CH = chloral hydrate.

**Figure 2:**
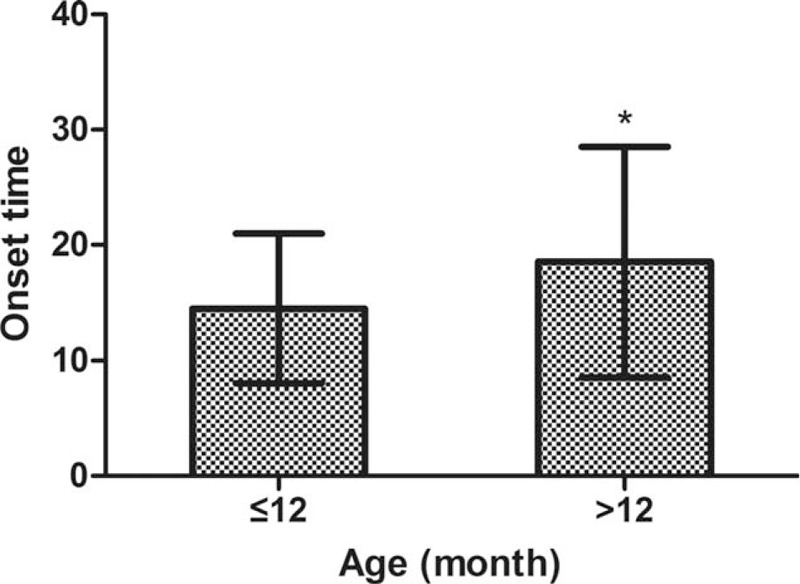
The onset time of rectal CH sedation in different age groups. There was a statistically significant difference between the groups of children ≤12 mo and >12 mo, ^∗^*P* < .05. CH = chloral hydrate.

The mean onset time was 16.41 (SD: 8.85) minutes, 57.3% of patients had a sedative effect at ≤15 minutes, and 92.4% of patients had a sedative effect at ≤30 minutes. The onset times and the corresponding proportions of patients are shown in Figure 1. When the children were grouped by age, the onset time was significantly different between the group of children ≤12 months and >12 months, (*P* < .05) (Fig. 2).

**Figure 3:**
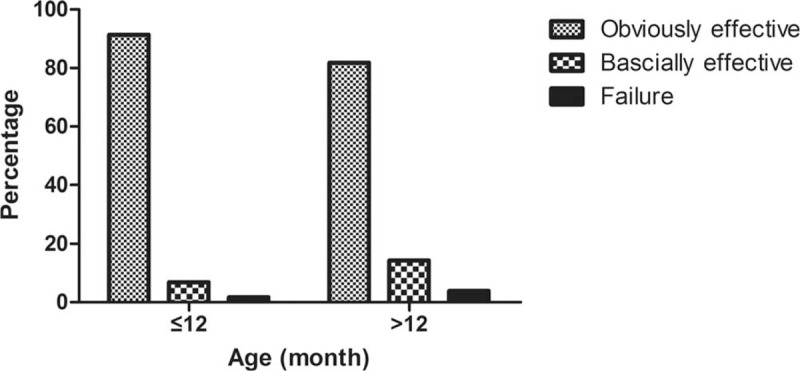
The sedative effect of rectal CH in different age groups. There was no statistically significant difference between the groups of children ≤12 mo and >12 mo. CH = chloral hydrate.

The CT scanning duration varied between 1 and 5 minutes, with a mean of 2.18 (SD: 0.82) minutes. The success rate, which was defined as the “obviously effective” and “basically effective” cases, was 97.0%. In the groups of children ≤12 months and >12 months, the success rates were 98.3% and 96.1%, respectively, and the failure rates were 1.7% and 3.9%, with no significant differences (Fig. 3).

There were 6 (4.4%) children who were not effectively sedated after the first dose, and 4 of them (3.0%) were also not effectively sedated even after supplementation with the second dose. These children were subsequently sedated with phenobarbital in 3 cases and diazepam in 1 case. Compared with the children who were effectively sedated after the first dose, the children who were not effectively sedated after the first dose were older (*P* < .01).

After sedation, the mean heart rate decreased by 11.9 per minute, the mean respiratory rate decreased by 6.3 per minute, and the mean oxygen saturation decreased by 0.2 (all *P* < .01, paired *t* test). All were within the normal limits, except 1 with transient hypoxia. These changes from before to after sedation are shown in Table 1.

**Table 1:** Changes in vital signs from before to after rectal CH sedation.

Characteristic	Before sedation	After sedation	*P*-value
Heart rate	122.50 ± 8.87	110.60 ± 7.10	<.001
Respiratory rate	33.44 ± 4.81	27.18 ± 1.84	<.001
Oxygen saturation	97.30 ± 1.32	97.05 ± 1.32	<.001

**Figure 4:**
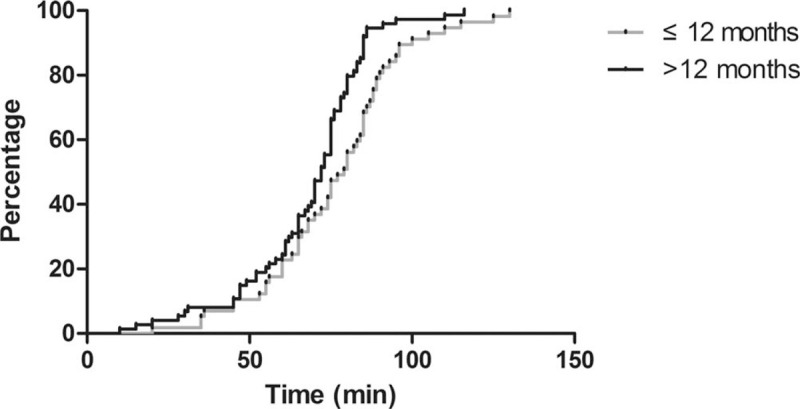
Recovery time of rectal CH sedation corresponding to the percentage of patients. CH = chloral hydrate.

The mean recovery time was 71.59 (SD: 20.60) minutes after CH administration. When compared, the recovery time was significantly different between the group of children ≤12 months and >12 months, (*P* < .05). The recovery following the time in each group is shown in Figure 4. The mean cost of CH sedation was 30.60 (SD: 3.19) RMB.

**Figure 5:**
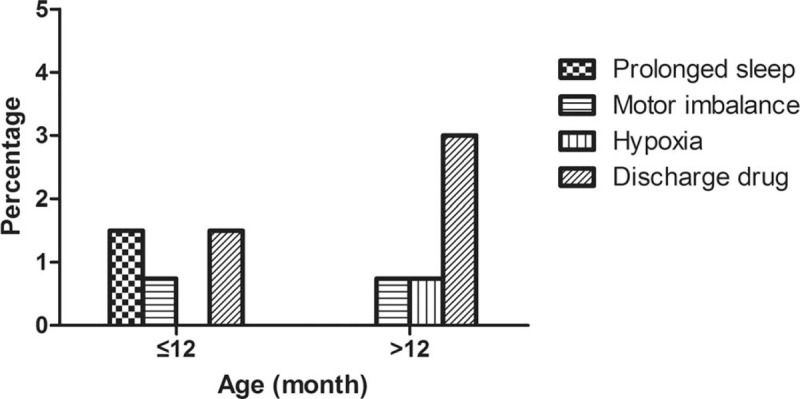
The adverse effect rate of rectal CH sedation in different age groups. CH = chloral hydrate.

In the study group, none of the children experienced CH-related vomiting; 6 (4.4%) patients discharged the rectal liquid early after administration; 2 (1.5%) patients experienced prolonged sleep (more than 2 hours after CH administration); 2 (1.5%) patients experienced motor imbalance; 1 (0.7%) patient experienced transient hypoxia that responded to repositioning and nasal catheter oxygen inhalation. The adverse effects in different age groups are shown in Figure 5. None of the children required further treatment of these adverse effects. These patients were monitored in the hospital until they resumed, and no further complications were found during follow-up.

Compared with another group of 62 cases with head trauma in our hospital who were previously administered oral CH for sedation, the results showed no significant differences in patient age and sex (*P* > .05), but the rectal CH group had a significantly shorter time to achieve sedation (*P* < .01) and a higher success rate (*P* < .01), although the difference in success rate was not significant in the ≤12 months subgroup (*P* > .05). There was no significant difference in recovery time between the 2 groups (*P* > .05). The detailed clinical data of the 2 groups are shown in Table 2. The adverse effect rate was significantly lower in the rectal CH group compared to that of the oral CH group (*P* < .01). The main reason was that there was no drug-related vomiting in the rectal CH group (*P* < .01) (Table 3).

**Table 2:** Comparison of the clinical data between the groups of rectal and oral CH sedation.

Items	Rectal CH (n = 135)	Oral CH (n = 62)	*P*-value
Age			
≤12 mo	9.24 ± 1.64	9.44 ± 2.04	.640
>12 mo	21.18 ± 6.07	20.92 ± 7.75	.358
Sex, M:F			
≤12 mo	35:23	14:11	.809
>12 mo	57:20	27:10	1.000
Onset time, min			
≤12 mo	13.65 ± 6.16	17.68 ± 3.40	.004
>12 mo	18.54 ± 9.99	24.83 ± 11.25	.003
Success rate, %			
≤12 mo	98.28	88.00	.080
>12 mo	96.10	81.08	.013
Recovery time, min			
≤12 mo	76.28 ± 21.58	79.77 ± 27.61	.554
>12 mo	67.97 ± 19.18	65.07 ± 27.24	.246

**Table 3:** Comparison of the adverse effects between the groups of rectal and oral CH sedation.

Adverse effects	Rectal CH (n = 135)	Oral CH (n = 62)	*P*-value
CH-related vomiting, n			
≤12 mo	0	6	.001
>12 mo	0	8	<.001
Prolonged sleep, n			
≤12 mo	2	2	.580
>12 mo	0	2	.103
Motor imbalance, n			
≤12 mo	1	1	.514
>12 mo	1	0	1.000
Hypoxia, n			
≤12 mo	0	1	.301
>12 mo	1	0	1.000
Discharge drug, n			
≤12 mo	2	0	1.000
>12 mo	4	0	.302

## 4 Discussion

CT scanning in children after head trauma is extremely important for establishing an early diagnosis in the ED, which requires a quiet, preferably sleeping patient, to minimize artifacts. Drug-induced sleep is especially required for those who are not cooperative with CT scanning. CH is a sedative hypnotic, and oral CH is the most common sedative method for nonpainful procedures in young children because of the high success rate.^[18–20]^ In our study, rectal CH was adopted to sedate the children for CT scanning, and the success rate was 97.0%, which was higher than that of oral administration. We think it is mainly due to the better cooperation of enema patients than oral patients. In our study, rectal CH showed the characteristics of rapid onset and complete recovery. Fast onset enables emergency patients get examination as soon as possible. Other examinations can also be performed within the duration of CH sedation, and a number of patients with multiple-site injuries underwent ultrasound examination after CT scanning in our study (data not shown). Thus, the duration of CH sedation allows for most emergency examinations, and we think it could have an important impact on emergency diagnosis. The difference in onset time and recovery time between age groups may be caused by the difference in metabolic capacity. Children who were not effectively sedated with the first dose of CH were older, so CH may work better in younger children.

Compared with oral CH group, the adverse effect rate was significantly lower in the rectal CH group. The main reason was that there was no drug-related vomiting in the rectal CH group. According to the literature, one of the most commonly reported adverse effects of oral CH is vomiting,^[12,21]^ and its incidence varies from 0.53% to 11.5%.^[22,23]^ In the rectal CH group, none of the children experienced drug-related vomiting, thus, rectal administration could avoid vomiting caused by oral administration. However, drug discharge was an adverse effect in rectal administration, as often occurs with other rectal medications.^[17]^ Nevertheless, CT scanning could not be carried out in only 2 cases, leading us to assume that the loss of medication was not significant.

Besides vomiting, other adverse effects occurred infrequently in our study. There were 2 cases of motor imbalance and 2 cases of prolonged sedation, and all these effects recovered spontaneously and required only supervision without any other interventions. We think these adverse effects are the result of incomplete metabolism of the drug.

Although CH was considered to have a minimal effect on respiration at the recommended therapeutic doses,^[8]^ brain injury is often combined with respiratory complications.^[24]^ In our study, 1 child experienced an episode of oxygen desaturation that resolved completely upon repositioning and nasal catheter oxygen inhalation. In our opinion, doctors and nurses should be in charge of the sedation and monitoring, and adequate ancillary support should be prepared in case of emergency.

No other serious adverse effects were noted in our study, confirming what studies have shown in recent years about CH: when administered in the proper dose, it has a good safety profile.^[25,26]^ In fact, CH has been used in infants with congenital heart disease with/without pulmonary infection in the pediatric cardiovascular intensive care unit.^[12]^

From our point of view, the advantage of rectal CH sedation was that we could benefit from the high success rate, safety, painless administration, no vomiting reaction, fewer adverse effects, and so on. Additionally, because of its economic benefits, it may be preferable in developing countries.

This study also had several limitations. In our study, most of the patients were infants and toddlers, so the effectiveness of CH in older children still needs further study. As a retrospective study, we did not detect the index of CH metabolism, for example, the rate of conversion to trichloroethanol, and its T 1/2. The optimal doses to achieve effective and risk-free CH still need to be determined, and it would be more useful to compare the effect of CH with those of other sedatives that could be used in ED conditions.

In summary, this research underscores the usefulness of rectal CH sedation for CT scanning in children with head trauma, given the ease of administration, effective sedation achieved, minimal effects on vital signs, and lack of serious adverse effects. When undertaken with proper monitoring and safety protocols in place, rectal administration of CH could be a practical and safe method of sedation for neurodiagnostic imaging in the ED. Wider use of CH in this field could mean easier, quicker, and more thorough examination in uncooperative patients.

In conclusion, we conclude that rectal CH is both effective and safe in sedating young children undergoing CT scanning in the ED, but only under specialized supervision.

## Author contributions

**Conceptualization:** Quanmin Nie.

**Data curation:** Peiquan Hui.

**Formal analysis:** Peiquan Hui.

**Investigation:** Peiquan Hui.

**Methodology:** Haitao Ding.

**Project administration:** Haitao Ding.

**Software:** Haitao Ding.

**Supervision:** Zengwu Wang.

**Writing – original draft:** Quanmin Nie.

**Writing – review & editing:** Zengwu Wang.
